# A Self-Powered and Highly Accurate Vibration Sensor Based on Bouncing-Ball Triboelectric Nanogenerator for Intelligent Ship Machinery Monitoring

**DOI:** 10.3390/mi12020218

**Published:** 2021-02-21

**Authors:** Taili Du, Xusheng Zuo, Fangyang Dong, Shunqi Li, Anaeli Elibariki Mtui, Yongjiu Zou, Peng Zhang, Junhao Zhao, Yuewen Zhang, Peiting Sun, Minyi Xu

**Affiliations:** 1 Marine Engineering College, Dalian Maritime University, Dalian 116026, China; dutaili@dlmu.edu.cn (T.D.); zuoxusheng@dlmu.edu.cn (X.Z.); dongfangyang@dlmu.edu.cn (F.D.); lishunqi443@dlmu.edu.cn (S.L.); mtyellie93@gmail.com (A.E.M.); zouyj0421@dlmu.edu.cn (Y.Z.); zhangpenglunji@dlmu.edu.cn (P.Z.); haoger@dlmu.edu.cn (J.Z.); 2 Collaborative Innovation Research Institute of Autonomous Ship, Dalian Maritime University, Dalian 116026, China

**Keywords:** self-powered, vibration sensor, triboelectric nanogenerator, machinery monitoring, intelligent ship

## Abstract

With the development of intelligent ship, types of advanced sensors are in great demand for monitoring the work conditions of ship machinery. In the present work, a self-powered and highly accurate vibration sensor based on bouncing-ball triboelectric nanogenerator (BB-TENG) is proposed and investigated. The BB-TENG sensor consists of two copper electrode layers and one 3D-printed frame filled with polytetrafluoroethylene (PTFE) balls. When the sensor is installed on a vibration exciter, the PTFE balls will continuously bounce between the two electrodes, generating a periodically fluctuating electrical signals whose frequency can be easily measured through fast Fourier transform. Experiments have demonstrated that the BB-TENG sensor has a high signal-to-noise ratio of 34.5 dB with mean error less than 0.05% at the vibration frequency of 10 Hz to 50 Hz which covers the most vibration range of the machinery on ship. In addition, the BB-TENG can power 30 LEDs and a temperature sensor by converting vibration energy into electricity. Therefore, the BB-TENG sensor can be utilized as a self-powered and highly accurate vibration sensor for condition monitoring of intelligent ship machinery.

## 1. Introduction

As we know, about 90% of world trade is carried by sea, which shows the great importance of sea transportation to human society. In recent years, with the development of artificial intelligence, big data, and the Internet-of-things technology, intelligent ship [1] has become an inevitable trend in the field of ship manufacturing and shipping industry. The development of intelligent ship is closely related to the intellectualization of main engine [2,3], generator diesel engine [4], air compressor [5], and other important machinery of ships. At the same time, the basis of intellectualization of the machinery is continuous and accurate working condition monitoring. It means that a large number of intelligent sensors are required. In addition, the condition monitoring of the mechanical equipment is primarily based on the working parameters, such as vibration [6,7], sound [8], temperature [9], pressure [10], flow rate [11], and so on. In particular, the key equipment on ships is most reciprocating or rotary machinery working at constant frequency in most instances. Additionally, because the vibration frequency can reflect the working condition of these machinery accurately and timely, a number of studies have been carried out on vibration monitoring. However, the previous research mainly focuses on vibration analysis [12,13,14] or fault diagnosis based on the vibration signals [15,16], rather than the vibration sensor itself. In addition, current commercial vibration monitoring sensors is mainly powered by marine electric power system through cables. Although the electrical power of ship can be self-sufficient, plenty of power and signal cables still increase the difficulty and cost of ship design and manufacturing. Especially, if the existing ships are to be intelligently retrofitted, it will be more difficult to break fireproof or watertight bulkheads and lay a huge number of additional cables. Moreover, due to long-term vibration, high temperature, and humidity of the ship machinery working environment, the ship signal cables are more likely to be damaged, which affects the work of the whole sensor network and even endangers the safety of the ship and crew. Therefore, the importance of finding a self-powered and highly accurate vibration sensor for the intelligent ships cannot be overemphasized.

The triboelectric nanogenerator (TENG), which is based on the coupling of triboelectric effect and electrostatic induction effect, was first proposed by Wang et al. [17] in 2012 and expected to be a promising power source or self-powered sensor [18]. Because of a low-cost, simple, and flexible structure, easy production and manufacture, high integrated level and high efficiency of the TENG [19], it has attracted the attention of many researchers around the world and has been applied to the energy harvesting in different fields [20], such as mechanical energy [21,22,23], wind energy [24], wave energy [25,26], acoustic energy [27], and human motion energy [28]. In addition, in order to collect different forms of energy more effectively, hybridized nanogenerators are also developed [29]. Furthermore, because the output voltage and current signals can represent the various dynamic motions triggered by mechanism, the TENGs can also be employed as a variety of self-powered sensors, such as vibration [30], wind speed [31], flow rate [32], displacement [33], pressure [34], tilt [35], and wave [36] sensors.

Especially in the application of TENG to vibration sensing, researchers have achieved certain research results in different fields. Hu et al. [37] designed a 3D spiral structure TENG with working bandwidth of 30 Hz, which showed potential applications in marine field. Chen et al. [38] proposed a harmonic-resonator-based TENG as an active sensor for ambient vibration detection. Wu et al. [39] developed a new pagoda-shaped TENG with the measurement range of 0–5 Hz and a measurement error less than 5% to the drilling downhole. Xu et al. [40] fabricated a spring-based TENG which can be used as a prime self-powered active vibration sensor to monitor the acceleration and frequency of the ambient excitation. Liu et al. [41] developed a self-powered and high sensitivity vibration sensor based on the established V-Q-a model, which provided a theoretical guidance for sensor designing and fabrication. Because of the resonant structure, these vibration sensors can always exhibit good output performance. However, their performance can also be limited at nonresonant frequency. At the same time, due to the use of spring or similar spring-based structures, it is usually complex and easy to be damaged, which also limits its reliability. Thus, on account of the advantages of real-time monitoring of the mechanical equipment working condition and broadband applicability of vibration sensor based on nonresonant structure TENG, it is also employed by researchers in different fields as well. Guo et al. [42] reported a TENG consisting of PTFE pellets for screw loosening and inclination monitoring of machinery. Zhang et al. [43] presented a sensitive acceleration sensor based on the TENG composed of mercury and PVDF film for mechanical equipment and human motion vibration monitoring. Zhang et al. [44] put forward a hybridized nanogenerator utilized as a vibrometer by combining TENG and electromagnetic-power-generation technology. Nevertheless, these vibration sensors are not suitable for the characteristics of tight installation space and frequent disassembly of vibration sensors on ship owing to their complexity, large size or difficulty in disassembly and assembly. Therefore, in this paper, the bouncing-ball triboelectric nanogenerator (BB-TENG) sensor is elaborately designed as a novel self-powered and highly accurate vibration sensor which can measure the vibration of the machinery on intelligent ships.

## 2. Composition and Working Principle of the BB-TENG Sensor

**Figure 1:**
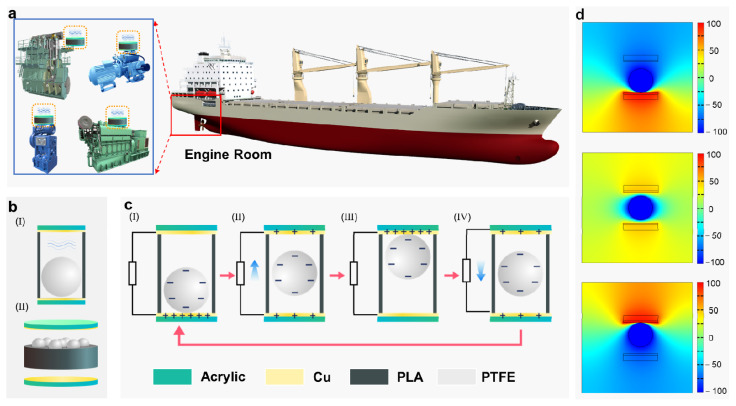
Composition and working principle of the bouncing-ball triboelectric nanogenerator (BB-TENG) sensor. (**a**) Schematic diagram of BB-TENG sensor applied to ship equipment vibration monitoring; (**b**) structure of the BB-TENG sensor; (**c**) working principle of the BB-TENG sensor; and (**d**) the electric potential distribution on the BB-TENG sensor by COMSOL Multiphysics^®^ (Version 5.5, COMSOL Inc., Stockholm, Sweden).

The arrangement of the BB-TENG sensor for monitoring the vibration of key equipment is shown in Figure 1a, which includes reciprocating machinery and rotary machinery, such as main diesel engines, generator diesel engines, air compressors, and reciprocating pumps on ship. Figure 1b shows the detailed structure of the BB-TENG sensor consisting of (1) polytetrafluoroethylene (PTFE) balls; (2) a circular poly lactic acid (PLA) container cylinder with holes arranged for the PTFE balls; (3) two acrylic plugs; and (4) two conductive copper foil electrodes. The PTFE balls inside the PLA container cylinders acts as the moving part of the sensor and the electronegative triboelectric layer of the TENG.

The working mechanism of the TENG is schematically demonstrated in Figure 1c. When a PTFE ball comes into contact with the copper electrodes, the copper electrodes will be positively charged, while the PTFE ball will be negatively charged due to different triboelectric polarities [45]. In the initial state, as shown in Figure 1c-(I), the contact between the PTFE ball and the bottom copper electrode (BCE) leads to the negative and positive charge on PTFE balls and copper electrode, respectively. When the vibration excitation of the external equipment outputs enough power to push the ball away from the BCE, an unbalanced potential difference will be generated between the top copper electrode (TCE) and BCE. As a result, electrons will be transferred from the TCE to the BCE through external circuit in order to balance the potential difference leading to an electric current from BCE to TCE, as demonstrated in Figure 1c-(II). It can be seen form Figure 1c-(III) that if the external excitation is large enough to drive the PTFE ball to contact with the TCE, the potential difference between the BCE and TCE is returned to equilibrium, and there is no current generated in the external circuit at this time. After that, under the combined action of gravity and counter-force of TCE, the PTFE ball goes downward. When it is apart from TCE, a new unbalance electric potential between TCE and BCE emerges. Therefore, as shown in Figure 1c-(IV), electrons move from BCE to TCE through an external circuit, creating an electric current opposite to the upward motion of the PTFE ball. Until the PTFE ball contacts with the BCE again, the working process backs to the initial state. The electric potential simulation result of the BB-TENG sensor by COMSOL Multiphysics^®^ with container cylinder height (*h*) of 10 mm and PTFE ball diameter (*d_b_*) of 8 mm is shown in Figure 1d. Apparently, the simulation results are in accordance with the above analysis. Thus, it can be observed that as long as there is enough external excitation, the continuous up and down motion of PTFE ball in PLA container cylinder will generate continuous electrical signal, which can reflect the vibration state of external excitation.

According to the theory of the contact-mode freestanding TENG, the governing equation for TENG can be written as:

(1) $$V=-\frac{1}{C}Q+V_{OC}=-\frac{d_{0}+g}{\varepsilon_{0}S}Q+\frac{2\sigma x}{\varepsilon_{0}}$$

where *C*, *Q*, *V_OC_*, *d*_0_, *g*, *S*, *ε*_0_, *σ* and *x* are the capacitance of the BB-TENG unit, transferred charge, the open-circuit voltage, the effective dielectric medium thickness, the total air-gap thickness between two electrodes, the effective contact area of the copper electrode layer, the dielectric constant in vacuum, the charge density on the PTFE ball surface, and the separation distance between the electrode layer and the PTFE ball surface respectively.

## 3. Experimental Section

### 3.1. Fabrication of the BB-TENG Sensor

In consideration of the quantified triboelectric serials [45] of common materials, the characteristics of BB-TENG sensor, the cost and availability of materials, PTFE, and copper foil, which have great difference in electronegativity, are selected as triboelectric materials of the BB-TENG sensor. Meanwhile, copper foil also acts as the electrode material of BB-TENG sensor. Referring to the results in [23] and considering the dimensions of the BB-TENG sensor, the device with the number of small balls of seven has been taken to carry out the experiments, which can not only meet the requirements of installation on ship machinery but also achieve a better signal-to-noise ratio.

In order to study the influence of ball diameter (*d_b_*) and PLA container cylinder size, especially the height of PLA container cylinder (*h*), on the performance of the BB-TENG, BB-TENG sensors composed of balls with different diameters are fabricated to conduct the experiments. The standard ball diameters of 5, 6.35, 7, 8, and 10.5 mm on the market are chosen considering the balance between signal-to-noise ratio and the size of the BB-TENG sensor. The PLA container cylinder height (*h*) of every diameter BB-TENG sensor is *d_b_* + 1, *d_b_* + 2, *d_b_* + 3, *d_b_* + 4 and *d_b_* + 5 mm, respectively. The model of the PLA (Cixi Lanbo Printing Consumables Co. Ltd., China) container cylinder is designed by the SolidWorks software. Then, the model is input to the Ultimaker Cura and printed by the 3D printer (Ultimaker 2 extended+, Ultimaker Holding B.V., Netherlands). The printing temperature is about 205 ℃, the infilling density is 80%, the printing speed is 30 mm/s, and the infilling form is a straight line. The corresponding PTFE balls (Shanghai Daoguan Rubber & Plastic Hardware Co., Ltd., Shanghai, China) are placed in to the PLA container holes. The PLA container cylinder is sealed up by the acrylic plugs, which are cut by the laser cut (LT-G-1530q, Liaocheng Longtai Laser Equipment Co., Ltd., Liaocheng, China). The size of the acrylic plug is adapting to the size of the PLA container. Two conductive copper foil electrodes (Shenzhen Wangxing Tape Co., Ltd., Shenzhen, China), which are self-adhesive type, are deposited to the internal surface of the acrylic plug. The electrodes have a fixed thickness of 0.03 mm. The size of the copper foil is equal to the internal surface of the acrylic plug. Copper wires, which is used to connect to the test equipment, are fixed to the copper foil. 

### 3.2. Experiment Setup

**Figure 2:**
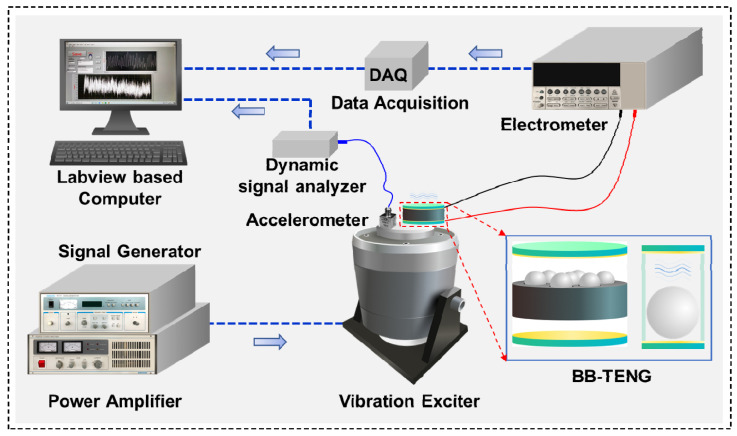
Apparatus for electrical signal test of the BB-TENG sensor.

Figure 2 indicates the test system for carrying out the working performance test. The test system is composed of signal generator (YE1311, SINOCERA, Suzhou, China), power amplifier (YE5872A, SINOCERA, Suzhou, China), vibration exciter (JZK-10, SINOCERA, China), electrometer (Keithley 6514, Tektronix, Beaverton, OR, USA), data acquisition device (cDAQ-9174, National Instruments, Cleveland, OH, USA), and LabVIEW (National Instruments, Cleveland, OH, USA) based computer. The signal generated by the signal generator is sent to the power amplifier. After the signal is amplified by the power amplifier, it is utilized to drive the moving plate of the vibration exciter to generate vibrational excitation. The signal generator can produce different types of signals according to different situations, and the sinusoidal signal is chosen considering the vibration characteristics of shipborne equipment. The equation of motion and the maximum acceleration of the vibration exciter, which can be got according to the calculus theory, is written as

(2) $$y_{b}=A\sin(\omega t+\varphi )$$

(3) $$a=A\omega^{2}=A{\left(2\pi f\right)}^{2}$$

where *y_b_* is the displacement distance of the inner surface of the BCE, *A* is the vibration amplitude, *ω* is the angular velocity, *t* is the transient time, *φ* is the initial phase angle, *a* is the maximum acceleration of the vibration exciter, and *f* is the vibration frequency of the vibration exciter.

The vibration frequency can be adjusted by the signal generator from 2 Hz to 2k Hz continuously, while the vibration amplitude depends on the power amplifier by adjusting the output voltage from 0 to 20 V DC. The BB-TENG sensor is firmly attached to the moving plate of the vibration exciter. With the vibration of the moving plate, PTFE balls in the BB-TENG sensor will move up and down. The continuous bouncing of PTFE balls between TCE and BCE can generate electrical signals such as open-circuit voltage (*V_oc_*), short-circuit current (*I_sc_*), and transferred charge (*Q*). For measuring the *V_oc_*, *I_sc_,* and *Q* of the BB-TENG sensor, and two copper electrodes are connected to the electrometer. The electrical signals are acquired by DAQ, then transferred to the LabVIEW based Computer. For the sake of verifying the performance of the BB-TENG sensor, an accelerometer (KS96.100, MMF, Radebeul, Germany) is also attached to the moving plate of the vibration exciter, and the signal generated by the accelerometer will be analyzed by the dynamic signal analyzer, and then transferred to the computer, so the amplitude of the vibration exciter can be determined. 

## 4. Results and Discussion

The fabricated BB-TENG sensors are tested by the test system at 5 Hz intervals over a range of 10 to 50 Hz. As shown in Table S1, it can be noticed from the statistics of different key equipment on different types of the ships that the vibration range of 10 to 50 Hz covers most vibration range of the machinery on ships. The vibration amplitude is determined to be in the range of 0.5 to 3 mm at intervals of 0.5 mm. Considering the vibration under abnormal working conditions, vibration amplitudes of 5 and 8 mm are also taken into account. It should be noted that the output of the vibration exciter is limited by the maximum amplitude, acceleration, and input current. As a result, some large amplitude and high frequency tests cannot be carried out. The working parameters of the vibration exciter is shown in Supplementary Table S2.

### 4.1. Vibration Frequency Sensing Characteristics

**Figure 3:**
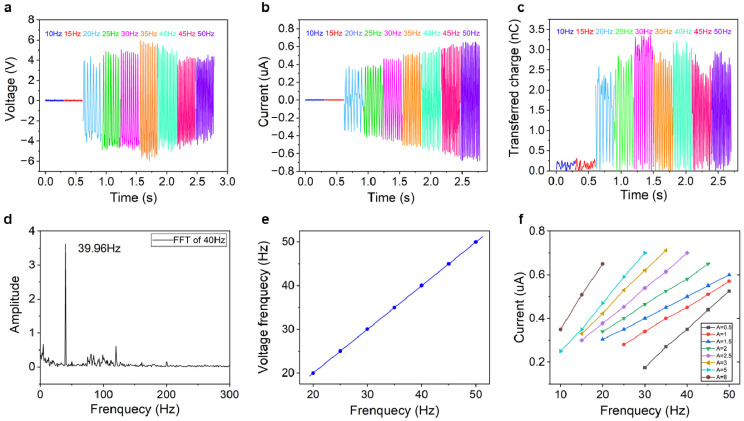
The vibration frequency monitoring characteristics of BB-TENG sensor with a ball diameter of 8 mm and container cylinder height of 10 mm. The (**a**) open-circuit voltage, (**b**) short-circuit current, and (**c**) transferred charge of BB-TENG sensor at different frequencies with a fixed vibration amplitude of 1.5 mm; (**d**) the FFT of the open-circuit voltage signal of the BB-TENG sensor with vibration frequency of 40 Hz; (**e**) the consistency between the vibration frequency and the FFT result of open-circuit voltage signal; and (**f**) relationships among the vibration frequency, vibration amplitude, and output short-circuit current of the BB-TENG sensor.

**Figure 4:**
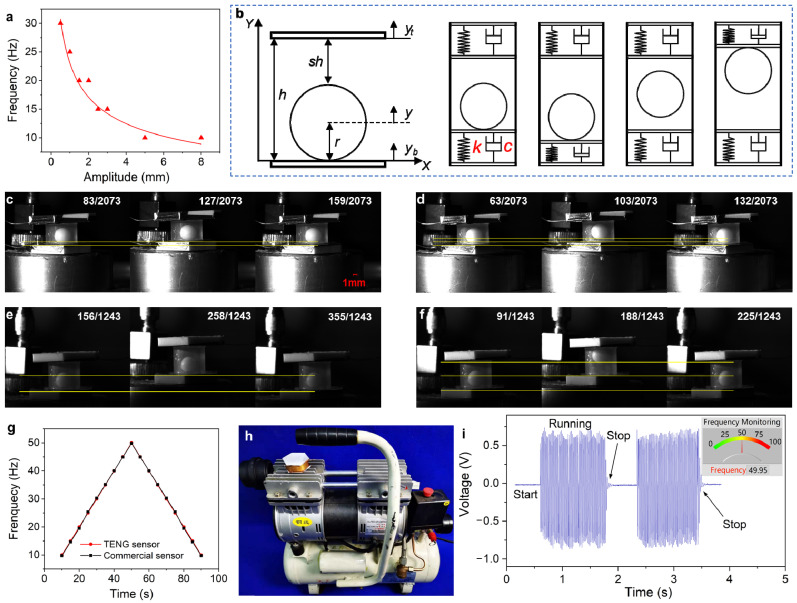
Working range and working characteristics of the BB-TENG sensor. (**a**) The minimum stable frequency at different amplitude. (**b**) Equivalent dynamic model of the PTFE ball bouncing in the container cylinder. PTFE ball-bouncing status at (**c**) frequency of 25 Hz, (**d**) frequency of 30 Hz and amplitude of 0.5 mm with a ball diameter of 5 mm and container cylinder height of 6 mm. PTFE ball-bouncing status at (**e**) frequency of 10 Hz, (**f**) frequency of 15 Hz, and amplitude of 3 mm with a ball diameter of 5 mm and container cylinder height of 8 mm. (**g**) Comparison between the BB-TENG sensor and a commercial vibration sensor. (**h**) The BB-TENG sensor is arranged on the air compressor and (**i**) used to monitoring the vibration condition of the air compressor.

For the reciprocating machinery or rotary machinery arranged on board ships, the working performance of them can be easily reflected by the dominant working frequency. For example, the main engine, which provides main propulsion power to propel the ship, has several cylinders. It can be imagined that all the cylinders produce equal power successively according to the firing sequence, and main working frequency can be stable in a certain value. However, in the case of abnormal working of one cylinder, the dominant working frequency changes and gives the sign that the main engine should be checked and repaired. Therefore, the vibration frequency sensing performance of the BB-TENG sensor is tested. On basis of the test result, the vibration dominant frequency monitoring characteristics of the BB-TENG sensor with a ball diameter of 8 mm and container cylinder height of 10 mm under different vibration amplitudes and frequencies are illustrated in Figure 3. The characteristics of *V_oc_*, *I_sc_*, and *Q* in the range of vibration frequency from 10 to 50 Hz with external excitation amplitude of 1.5 mm are shown in Figure 3a–c, respectively. It can be seen that the *V_oc_*, *I_sc_*, and *Q* are almost zero or highly volatile when the external vibration frequency is lower than 15 Hz. In the frequency range from 20 to 35 Hz, the *V_oc_* starts to increase from 8 V at 20 Hz, reaches the maximum 12 V at 35 Hz, and drops from 12 V to 8 V at 45 Hz gradually until it stabilizes at about 8 V at 50 Hz. The *I_sc_* increases from 0.3 μA at 20 Hz to 0.6 μA at 50 Hz showing a steady increasing trend. The change trend of *Q* is not very stable but within a relatively stable range. Although the variation tendency of the *V_oc_*, *I_sc_*, and *Q* are not consistent, it can be found that the frequency of the electrical signals after fast Fourier transform (FFT) is almost linear with the vibration frequency of the vibration exciter, as depicted in Figure 3e and Supplementary Figures S1 and S2. The correlation coefficient R^2^ is about 0.99 through correlation analysis. The FFT result is shown in Figure 3d. It can be seen that, when vibration frequency of vibration exciter is 40 Hz, the FFT of *V_oc_* is 39.96 Hz. Obviously, the error is less than 0.1%, showing a good vibration dominant frequency monitoring characteristic. In addition, the FFT results of *I_sc_* and *Q* are illustrated in Supplementary Figures S3 and S4. Therefore, based on the test results, the real-time vibration dominant frequency of the ship machinery can be obtained by doing fast Fourier transform of the BB-TENG sensor electrical signal, so as to acquire the real-time working condition of the machinery. Moreover, the BB-TENG sensor can also be used to measure low frequency vibration area such as 10 or 15 Hz with the vibration amplitude of 5 mm as shown in Figures S5–S7. It can be seen that the FFT of *V_oc_* is 9.99 and 15 Hz, when the vibration frequency of the vibration exciter is at 10 and 15 Hz, which shows high accuracy as well. Furthermore, when the PTFE ball cannot be bounced up, the open-circuit voltage is about 0.15 V as shown in Figure 4a, the signal-noise ratio (SNR) is equal to 20log(8/0.15) = 34.5 dB, which is very high. Therefore, the *V_oc_* should be taken as the original signal so as to enlarge the signal-noise ratio on account of its high signal output. 

Additionally, it can be discovered from Figure 3f that the change of *I_sc_* always shows a good linear relationship with the change of the vibration frequency in every specific amplitude, which reveals the other way to understand the vibration frequency or working condition of the ship machinery. In theory, according to the working principle of TENG [45] and the characteristics of PTFE ball, when the PTFE ball is charged by friction with copper electrode, the charge density on the surface of PTFE ball is constant for long time. The charge transfer between the two electrodes in external circuit occurs when the ball separates from one electrode until it comes into contact with the other. Because the charge transfer in each contact separation is consistent theoretically, and the relationship between short-circuit current and transferred charge is shown in the following equation:

(4) $$I=\frac{dQ}{dt}=\frac{Sd\sigma }{dt},$$

hence, the short-circuit current is only related to the number of contact separation between the ball and the upper and lower electrodes within a period of time. The shorter the contact-separation time, the greater the short-circuit current. The time of contact separation is related to the up and down movement frequency of the small ball in the container cylinder. Thus, the short-circuit current is related to the up and down movement frequency of the small ball. At the same time, as the frequency of the ball moving up and down is one-to-one corresponding to the frequency of the vibration exciter from 10 to 50 Hz with different amplitude, and the short-circuit current also shows a linear relationship with the frequency of the vibration exciter. 

### 4.2. Working Range of Vibration Frequency Sensing

With the assistance of the above analysis, it can be realized that the electrical signal is unstable and weak under low vibration frequency and low amplitude of the vibration exciter. Thus, those signals are not suitable to be used to check the dominant working frequency of the machinery. On the other hand, the electrical signal tends to be well balanced and be corresponded to the variation of the external vibration when the external vibration frequency and amplitude are higher than a certain value. According to the test results, just as illustrated in Figure 4a, with the increase of external vibration amplitude, the vibration frequency of initial output stable signal decreases. With different amplitude, the minimum stable frequency is from 10 to 30 Hz. When the vibration frequency is higher than the minimum stable frequency, the output signal will be stable up to the maximum frequency, 50 Hz, according to the test result and the videos filmed by the high-speed camera. Therefore, the detection limit of the BB-TENG is different with different vibration amplitude. The main reason for this phenomenon is that the characteristics of the electrical signal of the BB-TENG sensor is closely related to the bouncing status of the PTFE ball in the container cylinder. The stable output electrical signal can be achieved only when the bouncing status of the PTFE ball is stable and regular according to the theory of freestanding TENG [46].

In addition, the bouncing status of the PTFE ball in container cylinder under different external vibration excitation can be analyzed by Hertz’s theory [47]. The dynamical equation of the bouncing ball motion in the container cylinder can be derived directly by

(5) $$my^{\prime \prime }=F_{b}-F_{t}-mg,$$

where *m*, *y*, *F_b_*, *F_t_*, and *g* represent the mass of the PTFE ball, the distance of the PTFE ball in vertical direction, the impact force of the PTFE ball in contact with the bottom plug, and the impact force of the ball in contact with the top plug and gravitational acceleration (9.8 m/s^2^). *y*″ is the second derivative of *y*, which represents the acceleration of the ball. *F_b_* and *F_t_*, the impact forces between the bouncing ball and top or bottom plug, can be obtained through:

(6) $$F_{impact}=k\delta_{i}^{e}+cv_{i}.$$

According to the bouncing-ball motion model shown in Figure 4b, the following equations can be got:

(7) $$\begin{array}{l} F_{b}=\{\begin{array}{ll} -k{\left(y-r-y_{b}\right)}^{e}+c(y^{\prime }-y_{b}^{\prime }) & \;if\;y\leq y_{b}+r\\ 0 & \;else \end{array}\\ F_{t}=\{\begin{array}{ll} k{\left(y+r-y_{b}-h\right)}^{e}+c(y^{\prime }-y_{t}^{\prime }) & if\;y\geq y_{b}+h-r\\ 0 & else \end{array} \end{array}$$

where *r* is the radius of the bouncing ball, *y′* is the first derivative of *y* which represents the velocity of the ball, *y_b_′* is the first derivative of *y_b_*, which represents the velocity of the moving plate of the vibration exciter, *δ_i_* is the overlap of bouncing ball and plug when impacting, *v_i_* is the impact velocity, *k* is contact stiffness, *c* is the coefficient of contact damping, and *sh* is the space height between the top of the ball and the TCE.

The working process of the BB-TENG sensor has been demonstrated much more clearly by using high-speed camera (FATCAM Mini UX50, Photron, Tokyo, Japan) to make dynamic analysis of the bouncing status of the PTFE ball in the container cylinder. It can be seen from Figure 4c,e that the vibration energy is not enough to make the PTFE ball bouncing from the bottom plug with *d_b_* = 5 mm, *h* = 6 mm, *A* = 0.5 mm, *f* = 25 Hz and *d_b_* = 5 mm, *h* = 8 mm, *A* = 3 mm, and *f* = 10 Hz. At this time, the maximum acceleration is about 12.33 m·s^−2^ or 11.8 m·s^−2^. The bouncing-ball status is also shown in Supplementary Videos S1 and S2. Figure 4d,f show that the major bouncing status of the ball is in accordance with the vibration status of the vibration exciter under situation of *d_b_* = 5 mm, *h* = 6 mm, *A* = 0.5 mm, *f* = 30 Hz and *d_b_* = 5 mm, *h* = 8 mm, *A* = 3 mm, and *f* = 15 Hz, which are also demonstrated in Supplementary Videos S3 and S4. On the basis of the videos filmed by the high-speed camera, there are three important points to be noticed: (1) it can be known that even the maximum acceleration is larger than gravitational acceleration, and the PTFE ball may not be bounced by the vibration; (2) the Supplementary Videos S3 and S4 exhibit that the bouncing ball may be bounced twice or more when it contacts with the plugs, although it does not influence the monitoring accuracy; and (3) when the container height is 10 mm, the PTFE ball cannot be bounced to TCE all the time with *d_b_* = 5 mm, *h* = 10 mm, *A* = 0.5 mm, and *f* = 30 Hz, which shows chaos of the bouncing ball as displayed in Supplementary Video S5. Thus, it can be seen that if the container cylinder height is so high, the PTFE ball cannot bounce regularly over a wide vibration range. This is owing to the external vibration energy, the Poisson’s ratio, and Young’s modulus of the PTFE ball, acrylic plug, and copper electrode, and the bouncing status of the PTFE ball is also influenced by the coefficient of restitution and the collision duration [48], which will be discussed in our future work.

Based on the analysis from the above test results, for the sake of enlarging the applicable vibration frequency monitoring range of the BB-TENG sensor, the height of container cylinder of the device should be made as small as possible. In this work, the BB-TENG sensor with *d_b_* = 7 mm, *h* = 8 mm is determined to be employed to the vibration condition monitoring of an air compressor. It can be seen from Figure 4g that, comparing with a commercial vibration sensor, when the external vibration frequency changes from 10 to 50 Hz and then from 50 to 10 Hz, there is almost no difference between them. Meanwhile, mean detection error of the BB-TENG sensor is less than 0.05%, which shows high accuracy of the BB-TENG sensor. As exhibited in Figure 4h, the BB-TENG sensor is capable of monitoring the start, running, and stop status of the air compressor, which proves its potential to be an accurate vibration sensor. The air compressor with two pistons and two cylinders works at about 1500 rpm, and each cylinder works one time in one revolution; therefore, the working frequency of it is 1500 × 2/60 = 50 Hz. The vibration frequency detected by the BB-TENG sensor, 49.95 Hz, is demonstrated in the inset of Figure 4h. It is almost the same with the actual vibration frequency of the air compressor, which exhibits good performance on the vibration frequency monitoring of machinery.

### 4.3. Self-Powered Performance

**Figure 5:**
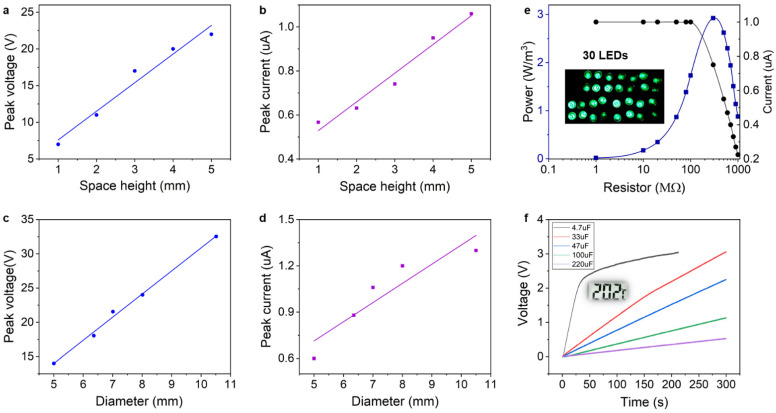
The power generation performance of the BB-TENG. (**a**) Maximum open-circuit voltage and (**b**) maximum short-circuit current of the BB-TENG with a ball diameter of 7 mm at different container cylinder height. (**c**) Maximum open-circuit voltage and (**d**) maximum short-circuit current of the BB-TENG with different ball diameter. (**e**) Output power and current of the BB-TENG in series with different load resisters and (**f**) different capacitors charged by BB-TENG with ball diameter of 10.5 mm.

Except for performance of sensing, the BB-TENG sensor also shows the potential to be self-powered by means of collecting the vibration energy of the ship machinery and converting it to the electric energy. In order to explore the self-powered potential of the BB-TENG, the power generation performance of it is shown in Figure 5. Figure 5a,b depict the relationships among the maximum *V_oc_* and *I_sc_* of the BB-TENG with the same bouncing ball (*d_b_* = 7 mm) and different container cylinder height. With the increase of the container cylinder height, the maximum *V_oc_* at different container cylinder height increases as well. The maximum *V_oc_* and *I_sc_* reach about 22 V and 1.06 μA, respectively, when the container cylinder height is 5 mm. Additionally, the maximum *V_oc_*, and *I_sc_* of the BB-TENG with different PTFE ball diameter has been shown in Figure 5c,d. Obviously, the maximum *V_oc_* and *I_sc_* of the BB-TENG increases along with the increase of the PTFE ball diameter until they rise to 32.5 V and 1.3 μA, respectively. Therefore, when the BB-TENG is applied to be the energy harvester on condition that the vibration energy and installation space is enough, we should choose these two parameters as large as possible.

According to the test results and the actual vibration conditions of the ship machinery, the output power of the BB-TENG with *d_b_* = 10.5 mm, *h* = 15.5 mm, *f* = 35 Hz, and *A* = 2.5 mm has also been studied by changing different resistors in series. As depicted in Figure 5e, the maximum output power density is about 3 W/m^3^ with the resistor of 300 MΩ. Moreover, 30 LEDs can be lit up as shown in the inset of Figure 5e and Supplementary Video S6. Furthermore, different capacitors are chosen to be charged by the BB-TENG, and the charging process of 4.7 μF, 33 μF, 47 μF, 100 μF, and 220 μF capacitors is shown in Figure 5f. Thus, the larger the capacitor is, the lower the charging rate is. The 4.7 μF capacitor can be charged to 2.2 V quickly in 35 s and then the charging rate slows down, rising from 2.2 V to 3 V in 160 s due to the increasing capacitive reactance with the increasing voltage. In addition, the voltage of the 33 μF capacitor can achieve 3 V after charging for 590 s, and it is able to power a temperature sensor. As it has been illustrated in Figure 5f, BB-TENG has great potential in series to be a power source of the distributed sensor network.

## 5. Conclusions

In this study, a self-powered and highly accurate BB-TENG sensor for intelligent ship machinery vibration monitoring is proposed and fabricated. The BB-TENG sensor consists of two acrylic plugs, one container cylinder, two copper electrodes attached to the inner surface of the plugs, and PTFE bouncing balls. The effects of PTFE ball diameter, container cylinder height, and vibration conditions on the electrical performance of the BB-TENG has been systematically studied. It is found that the short-circuit current, as well as the fast Fourier transform of electrical signals including the open-circuit voltage, short-circuit current, and transferred charge of the BB-TENG sensor agrees well with the dominant external vibration frequency. As demonstrated experimentally, the BB-TENG sensor has a high signal-to-noise ratio of 34.5 dB with mean error less than 0.05% at the vibration frequency of 10 to 50 Hz, which covers the most vibration range of the machinery on ship. In addition, minimum stable frequency and vibration acceleration have been verified by employing high-speed camera, which provides a prospective method for selecting suitable BB-TENG sensor due to different vibration characteristics of different ship machinery. The starting, running, and stop status and the working frequency of the air compressor is well sensed by the BB-TENG sensor. Moreover, by researching power generation characteristics of BB-TENG, it is found to be able to light up 30 LEDs and power the temperature sensor after charging the capacitor, which shows the promising potential to be a sustainable power source. Notably, comparing with other commercial vibration sensors, the BB-TENG sensor not only has high accuracy but also can be self-powered. Thus, it is obvious that the BB-TENG sensor has great potential application in condition monitoring of intelligent ship machinery.

## Data Availability

The data presented in this study are available on request from the corresponding author.

## Supplementary Materials

The following are available online at https://www.mdpi.com/2072-666X/12/2/218/s1, Table S1: working frequency of different key equipment on different types of ships, Table S2: working parameters of the vibration exciter, Figure S1: the consistency between the vibration frequency and the FFT result of short-circuit current, Figure S2: the consistency between the vibration frequency and the FFT result of transferred charge signal, Figure S3: the FFT of the short-circuit current signal of the BB-TENG sensor, Figure S4: the FFT of the transferred charge signal of the BB-TENG sensor, Figure S5: Open-circuit voltage of BB-TENG sensor at different frequencies with a fixed vibration amplitude of 5 mm, Figure S6: The FFT of open-circuit voltage signal of BB-TENG sensor with vibration frequency of 10 Hz, Figure S7: The FFT of open-circuit voltage signal of BB-TENG sensor with vibration frequency of 15 Hz, Video S1: PTFE ball-bouncing status with *f* = 25 Hz, *A* = 0.5 mm, *d_b_* = 5 mm, and *h* = 6 mm, Video S2: PTFE ball-bouncing status with *f* = 10 Hz, *A* = 3 mm, *d_b_* = 5 mm, and *h* = 8 mm, Video S3: PTFE ball-bouncing status with *f* = 30 Hz, *A* = 0.5 mm, *d_b_* = 5 mm, and *h* = 6 mm, Video S4: PTFE ball-bouncing status with *f* = 15 Hz, *A* = 3 mm, *d_b_* = 5 mm, and *h* = 8 mm, Video S5: PTFE ball-bouncing status with *f* = 30 Hz, *A* = 0.5 mm, *d_b_* = 5 mm, and *h* = 10 mm, Video S6: 30 LEDs lit up by the BB-TENG sensor.
