# Supplementary material for: A Self-Powered and Highly Accurate Vibration Sensor Based on Bouncing-Ball Triboelectric Nanogenerator for Intelligent Ship Machinery Monitoring
Source: Micromachines (Basel). 2021 Feb 21;12(2):218. doi: 10.3390/mi12020218 (PMC7927136; doi:10.3390/mi12020218)
Supplement: Supplementary file 1 [file micromachines-12-00218-s001.zip › micromachines-1104735-supplementary/Supporting information-final version .pdf]

micromachines

MDPI

Supplementary Materials

A Self-powered and Highly Accurate Vibration Sensor Based on Bouncing Ball Triboelectric Nanogenerator for Intelligent Ship Machinery Monitoring

Taili Du, Xusheng Zuo, Fangyang Dong, Shunqi Li, Anaeli Elibariki Mtui, Yongjiu Zou, Peng Zhang, Junhao Zhao, Yuewen Zhang, Peiting Sun and Minyi Xu

Table S1. Working Frequency of different key equipment on different types of ships

No.	Ship Type	Equipment	Equipment type	Power (kW)	Number of cylinders	Revolution Speed (rpm)	Working Frequency (Hz)
1	Container ship 14000TEU[1] LOA[2]: 366 m W[3]: 51.2 m H[4]: 29.9 m	Main engine	12K98ME-C	72240	12	104	20.8
		Generator set	8L32/40	4000	8	720	48
		Air compressor	WP 271L	52	3 stage	1780	29.67 / cylinder
		Emergency generator	NTA855DMGE	300	6	1800	90
		Refrigeration unit	CRKC	6.3	2	1250	41.67
2	Bulk Carrier 57,000 dwt[5] LOA: 182.98 m W: 32.26 m H: 17.15 m	Main engine	6S50MC-C	9480	6	127	12.7
		Generator set	DK520E	420	5	900	37.5
		Air compressor	H-74	30	2 stage	1200	20 / stage
		Emergency generator	D0226MTE	98	6	1800	90
		Refrigeration unit	FX-4	5.5	4 (2 set)	920	15.33
3	General Cargo Ship 30,000 dwt LOA: 199.80 m W: 27.80 m H: 15.50 m	Main engine	6RT-flex58T-D	10850	6	105	10.5
		Generator set	1050W6L20	1260	6	900	45
		Air compressor	XW-150	31	2 stage	1775	29.6 / cylinder
		Emergency generator	TBD234V6	244	6	1800	90
		Refrigeration unit	4G.2Y	21.3	4 (2 set)	1750	29.17
4	Training Ship 6600 GT[6] LOA: 116.00 m W: 18.00 m H: 8.35 m	Main engine	6S35MC Mk7	4440	6	173	28.83
		Generator set	520W4L20	545	4	1000	33.33
		Air compressor	HV2/200	22	2 stage	725	12.08 /c ylinder
		Emergency generator	TBD234V8	249	8	1500	100
		Refrigeration unit	4H.2Y	15.6	4 (2 set)	1450	24.17
5	Tug 2850 GT LOA: 59.20 m W: 14.00 m H: 6.00 m	Main engine	6MG28HZ	1838	6	750	37.5
		Generator set	KMTA	403	6	1000	50
		Air compressor	2ZS-0.34/3-BS	5	2 stage	600	10 / cylinder
		Emergency generator	/	/	/	/	/
		Refrigeration unit	2CC-3.2Y-40S	2.65	2	1450	48.33

[1] TEU means twenty-foot equivalent unit;

[2] LOA means Length overall, overall length of the ship;

[3] W means the breadth moulded, maximum breadth of the ship measured inside the inner shell strakes of plating, and usually occurs amidships;
[4] H means the depth moulded, vertical distance between the moulded base line and the top of the beams of the uppermost continuous deck measured at the side amidships of the ship;
[5] dwt means deadweight tonnage, a measure of how much weight a ship can carry;
[6] GT means gross tonnage, calculated based on "the moulded volume of all enclosed spaces of the ship".

There are 5 different types of ships are listed in Table S2 including container ship, bulk carrier, general cargo ship, training ship and special purpose ship. The key equipment including main engine, generator set, air compressor, emergency generator and refrigeration unit made by different mainstream manufactures are also indicated in Table S2. It can be seen from Table S2 that the vibration frequency of most key equipment is from 10 Hz to 50 Hz except the emergency generator. It should be noted that the emergency generator is only operated for short time in event of emergency situations rather than long period continuous running as other key equipment, so it is unnecessary to keep continuous monitoring of it. In addition, the vibration frequency can be easily calculated through

$$f_e = \frac{n \cdot r}{60},$$

where, f_e is the vibration frequency of the equipment, n is the number of the cylinder, and r is the revolution speed. However, the generator set are most four stroke diesel engine, which means each cylinder works one time every two revolutions. Moreover, the main engine on some small ships such as the No.5 (Tug) is also four stroke diesel engine. So, the vibration frequency of the generator set should be calculated by

$$f_e = \frac{n \cdot r}{60 \times 2}.$$

In addition, in order to compress the air to about 30MPa for starting the main engine, the compression process of an air compressor generally has 2 or 3 stages. Different stage has different working characteristics, so the vibration frequency should be monitored separately.

Table S2. Working parameters of the vibration exciter

Model	JZK-10
Maximum vibration force (N)	100
Maximum amplitude (mm)	±10
Maximum input current (Arms)	≤10
Maximum acceleration (g)	10
Frequency range (Hz)	DC-2K
DC coil resistance (Ω)	0.7
Force constant	10
Effective moving mass(Kg)	0.35
Output type	Mandril

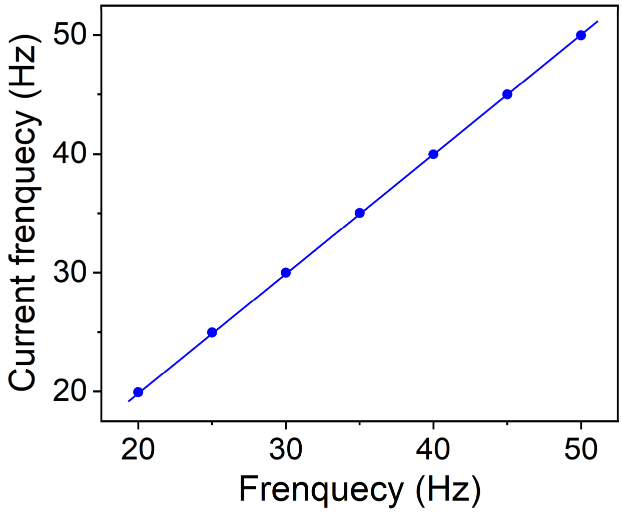

Figure S1. The consistency between the vibration frequency and the FFT result of short-circuit current signal

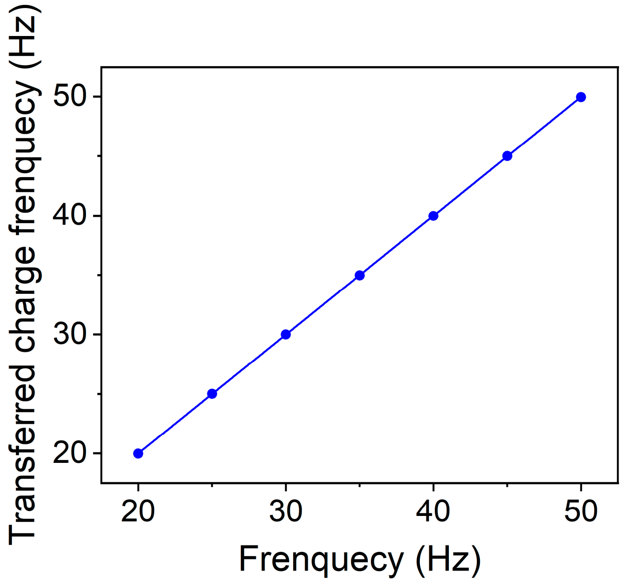

Figure S2. The consistency between the vibration frequency and the FFT result of transferred charge signal

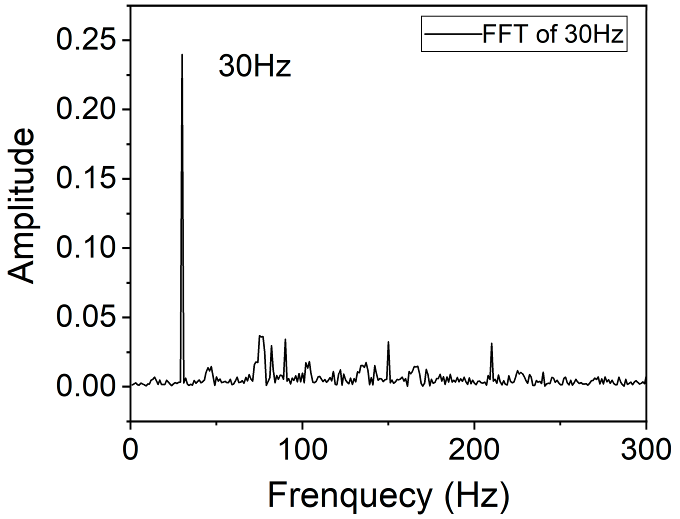

Figure S3. The FFT of the short-circuit current signal of the BB-TENG sensor with vibration frequency of 30Hz

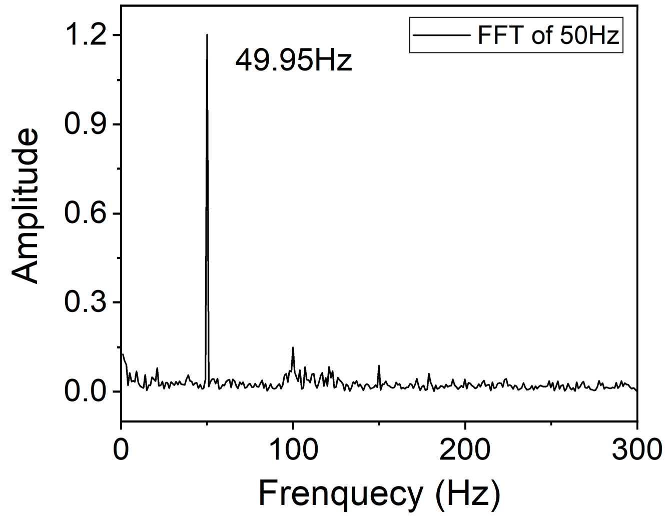

Figure S4. The FFT of the transferred charge signal of the BB-TENG sensor with vibration frequency of 50Hz

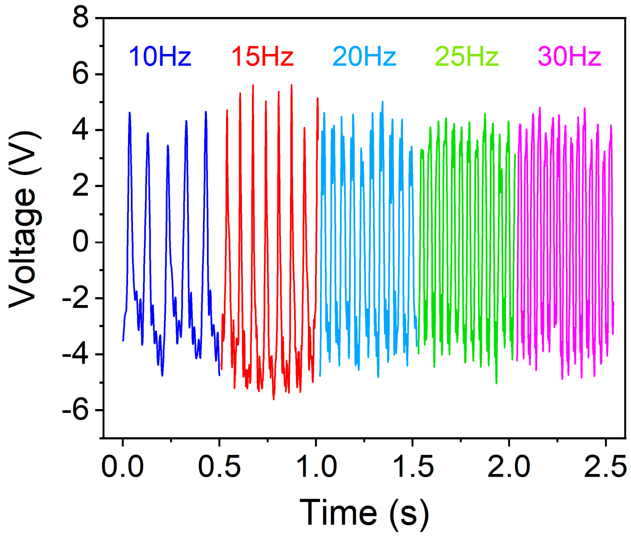

Figure S5. Open-circuit voltage of BB-TENG sensor at different frequencies with a fixed vibration amplitude of 5mm

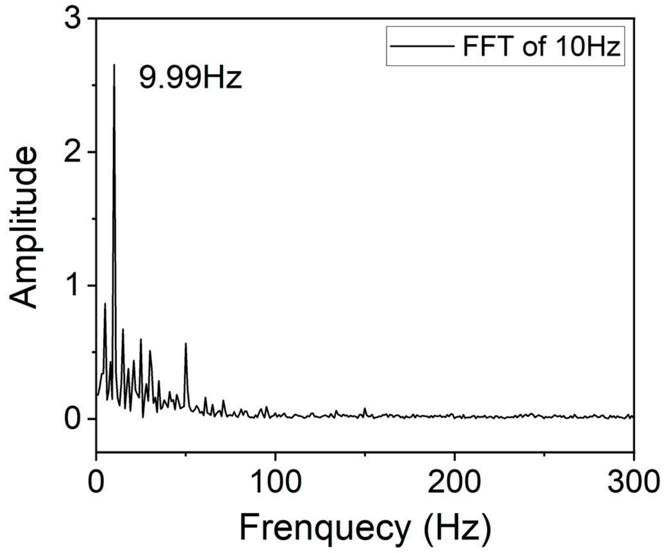

Figure S6 The FFT of open-circuit voltage signal of BB-TENG sensor with vibration frequency of 10 Hz

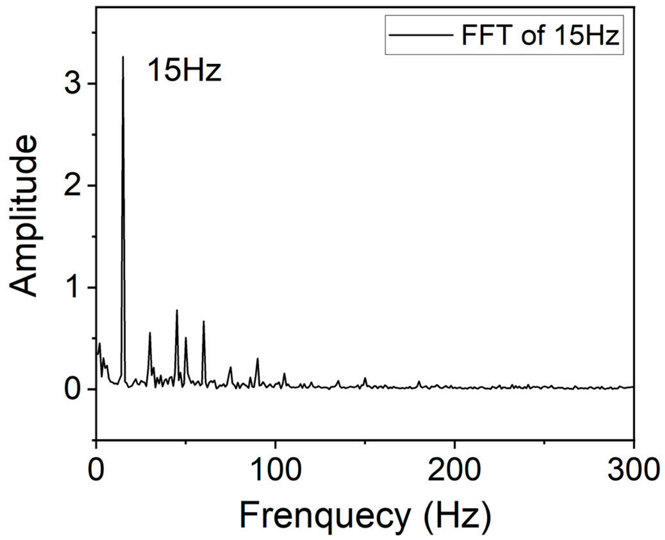

Figure S7 The FFT of open-circuit voltage signal of BB-TENG sensor with vibration frequency of 15 Hz

Supplementary Video S1: PTFE ball bouncing status with f=25 Hz, A=0.5 mm, d_b=5 mm, and h= 6mm

Supplementary Video S2: PTFE ball bouncing status with f=10 Hz, A=3 mm, d_b=5 mm, and h=8 mm

Supplementary Video S3: PTFE ball bouncing status with f=30 Hz, A=0.5 mm, d_b=5 mm, and h=6 mm

Supplementary Video S4: PTFE ball bouncing status with f=15Hz, A=3mm, d_b=5mm, and h=8mm

Supplementary Video S5: PTFE ball bouncing status with f=30 Hz, A=0.5 mm, d_b=5 mm, and h=10 mm

Supplementary Video S6: 30 LEDs lit up by the BB-TENG sensor
